# Genetic and functional interaction network analysis reveals global enrichment of regulatory T cell genes influencing basal cell carcinoma susceptibility

**DOI:** 10.1186/s13073-021-00827-9

**Published:** 2021-02-06

**Authors:** Christelle Adolphe, Angli Xue, Atefeh Taherian Fard, Laura A. Genovesi, Jian Yang, Brandon J. Wainwright

**Affiliations:** 1 Institute for Molecular Bioscience, The University of Queensland, Brisbane, QLD 4072 Australia; 2 The University of Queensland Diamantina Institute, Translational Research Institute, Woolloongabba, QLD 4102 Australia; 3 Australian Institute for Bioengineering and Nanotechnology, The University of Queensland, Brisbane, QLD 4072 Australia; 4 School of Life Sciences, Westlake University, Hangzhou, 310024 Zhejiang China; 5 Westlake Laboratory of Life Sciences and Biomedicine, Hangzhou, 310024 Zhejiang China

**Keywords:** BCC, GWAS, Cancer susceptibility, Immune surveillance, Protein interaction networks

## Abstract

**Background:**

Basal cell carcinoma (BCC) of the skin is the most common form of human cancer, with more than 90% of tumours presenting with clear genetic activation of the Hedgehog pathway. However, polygenic risk factors affecting mechanisms such as DNA repair and cell cycle checkpoints or which modulate the tumour microenvironment or host immune system play significant roles in determining whether genetic mutations culminate in BCC development. We set out to define background genetic factors that play a role in influencing BCC susceptibility via promoting or suppressing the effects of oncogenic drivers of BCC.

**Methods:**

We performed genome-wide association studies (GWAS) on 17,416 cases and 375,455 controls. We subsequently performed statistical analysis by integrating data from population-based genetic studies of multi-omics data, including blood- and skin-specific expression quantitative trait loci and methylation quantitative trait loci, thereby defining a list of functionally relevant candidate BCC susceptibility genes from our GWAS loci. We also constructed a local GWAS functional interaction network (consisting of GWAS nearest genes) and another functional interaction network, consisting specifically of candidate BCC susceptibility genes.

**Results:**

A total of 71 GWAS loci and 46 functional candidate BCC susceptibility genes were identified. Increased risk of BCC was associated with the decreased expression of 26 susceptibility genes and increased expression of 20 susceptibility genes. Pathway analysis of the functional candidate gene regulatory network revealed strong enrichment for cell cycle, cell death, and immune regulation processes, with a global enrichment of genes and proteins linked to T_Reg_ cell biology.

**Conclusions:**

Our genome-wide association analyses and functional interaction network analysis reveal an enrichment of risk variants that function in an immunosuppressive regulatory network, likely hindering cancer immune surveillance and effective antitumour immunity.

**Supplementary Information:**

The online version contains supplementary material available at 10.1186/s13073-021-00827-9.

## Background

Basal cell carcinoma (BCC) is the most common form of human cancer, with more than 90% of tumours presenting with genetic activation of the Hedgehog (HH) pathway [1]. The current model of BCC development is that cumulative sun exposure induces characteristic ultraviolet (UV) signature mutations, resulting in DNA damage within basal cells of the skin [2]. Individuals at highest risk of developing BCC are those with fair skin, blonde hair, red hair, and pale coloured eyes [3, 4], predominantly due to decreased photoprotection (the absorption of UV photons and reactive oxygen species provided by melanin pigment) [5]. Greater than 99% of BCC cases arise sporadically, without a clear inheritable disease-causing mutation, highlighting the impact that both environmental factors and the sum of an individuals’ genetic variation play in determining whether driver mutations, such as the presence of HH pathway activating mutations, culminate in BCC development. This is most clearly evidenced in the many “BCC-prone” individuals who have no evidence of a monogenic germline predisposition.

Genome-wide association studies (GWAS) have played a key role in identifying the polygenic effects that confer susceptibility to BCC. Loci have been attributed to a wide variety of biological processes including photoprotection, cellular trafficking, cytoskeletal organisation, cell motility/migration, skin biology, ectoderm/mesoderm differentiation, cell death, telomere biology, immune, tumour progression, DNA repair, and cell cycle regulation [6–12]. Although GWAS provide a framework for identifying putative susceptibility loci, they rarely identify causal genes, predominantly due to the complicated linkage disequilibrium (LD) structure of the genome, in addition to the fact that genetic variants can affect phenotype via distant regulation of gene expression. To circumvent this problem, several statistical methods have been developed to prioritise functionally relevant genes from GWAS loci [13–17], including the Summary-data-based Mendelian Randomisation (SMR) and HEterogeneity In Dependent Instruments (HEIDI) tests. The SMR and HEIDI methodology [16] combines summary-level GWAS data and expression quantitative trait locus (eQTL) studies to identify whether a transcript and phenotype are associated because of a single and/or set of shared causal variant(s), thereby identifying functionally relevant candidate genes. An emerging area expanding on current methods of GWAS data analyses involves production of network annotations that represent functional interactions among genes and their products. Network-assisted analysis allows advanced analyses of the associated loci and/or candidate genes by assessing the combined effects of multiple genes participating in a network, thereby providing a global view of the genetics underlying a particular human disease or trait.

Here, we describe an integrative analysis of summary statistics from GWAS, eQTL, and methylation quantitative trait locus (mQTL) studies culminating in the construction of two functional interaction (FI) networks underlying BCC susceptibility. We have been able to identify previously reported GWAS hits as functional candidate genes by demonstrating a direct correlation between GWAS SNP association and changes in gene expression. Subsequent network analysis revealed a strong enrichment of immune regulatory genes, revealing genetic susceptibility to BCC is profoundly influenced by inherited background immune traits.

## Methods

### Genome-wide association study

Initial quality control (QC) and imputation of the genotype data on Haplotype Reference Consortium (HRC) [18] panel were carried out by the UK Biobank [19]. We performed further QC (excluding SNPs with minor allele count < 5, Hardy-Weinberg equilibrium test *P* value < 1 × 10^−6^, missing genotype rate > 5%, or imputation info score < 0.3) using PLINK2 [20]. BCC cases consisted of (1) BCC (UK Biobank data field ID: 1061) from self-reported cancers (UK Biobank data field ID: 20001) and (2) BCC defined by the histology of cancer tumour (UK Biobank data field ID: 40011) within cancer registry records (field ID: 40006). Controls were individuals without any self-reported cancer or cancer registry record. Detailed gender and age demographics of the 17,416 cases and 375,455 controls are represented in Additional file 1: Figure S1. GWAS analysis was performed using BOLT-LMM [21] with fitting gender, age, and first ten principal components (PCs) as the covariates. We included ~ 700,000 SNPs obtained by LD pruning (*r*^2^ < 0.9) from HapMap3 SNPs as “model SNPs” in the BOLT-LMM analysis to adjust for relatedness, population structure, and polygenicity. The beta estimates from BOLT-LMM were transformed on the binary phenotype to the odds ratio (OR) scale by LMOR [22]. The index SNPs are clumped based on *P* < 5 × 10^−8^, a 1-Mb window, and a LD *r*^2^ threshold of 0.01. The SNP-based heritability was estimated by LD score regression (LDSC) [23]. When estimating the heritability in liability scale, the sample prevalence and population prevalence were both set as 4.43%. Conditional and joint association analysis (COJO) [24] was conducted based on a stepwise selection model to identify a set of jointly associated (and near-independent) SNPs. Loci were classified as novel if located outside a 1-Mb window of previously reported GWAS hits (GWAS Catalog database [25]).

### Summary-data-based Mendelian Randomisation analysis

SMR and HEIDI analyses were conducted as previously described [16] (SMR software: http://cnsgenomics.com/software/smr/). In brief, the SMR method selects the top eQTL SNP as an instrumental variable to estimate the effect of the gene expression on the trait of interest (in the Mendelian randomisation framework). Selection criteria include *cis*-eQTL SNPs located within a 2-Mb window of the target gene probe with *P*_eQTL_ < 5 × 10^−8^. The HEIDI filtering test adopts multiple SNPs in a *cis*-eQTL region to reject the significant SMR associations due to LD between disease-associated SNPs and eQTL SNPs. The eQTL summary statistics were obtained from the eQTLGen Consortium (*n* = 31,684 blood samples) and GTEx dataset (GTEx Portal: https://gtexportal.org/home/index.html; *n* = 369 whole blood sample, *n* = 605 sun exposed lower leg, *n* = 517 sun not exposed suprapubic). The gene expression levels were measured using Illumina gene expression arrays, and the genotype was imputed to 1KGP [26]. The mQTL summary data were generated from a genetic analysis of DNA methylation measured on Illumina HumanMethylation450 arrays (*n* = 1980 in peripheral blood) [27]. The statistical power of SMR analysis has been demonstrated by simulation in a previous study [28] implementing the SMR workflow used in this study.

### Functional interaction networks

Functional interaction networks were constructed using the ReactomeFIViz App (ReactomeFIViz app and Reactome FI Network, Wu and Haw 2017 PMID: 28150241) in Cytoscape (v3.6.1) [29]. GWAS-FI network was constructed using nearest genes to each of the 71 GWAS loci. SMR FI network was constructed using the 46 eQTLGen derived SMR genes. Pathway-enrichment analysis was performed within the ReactomeFIViz app. ReactomeFIViz utilises a comprehensive protein functional interaction network construed from the integration of multiple external data resources including protein-protein interaction networks of several organisms (including human and mouse) in addition to biological pathway databases such as KEGG and Reactome [30]. The information gathered from these resources is served as training data for a Naïve Bayes Classifier, which is ultimately used to predict and annotate functional interaction network for a given gene set [31].

## Results

### GWAS identifies 3 previously undescribed BCC susceptibility loci

**Fig. 1:**
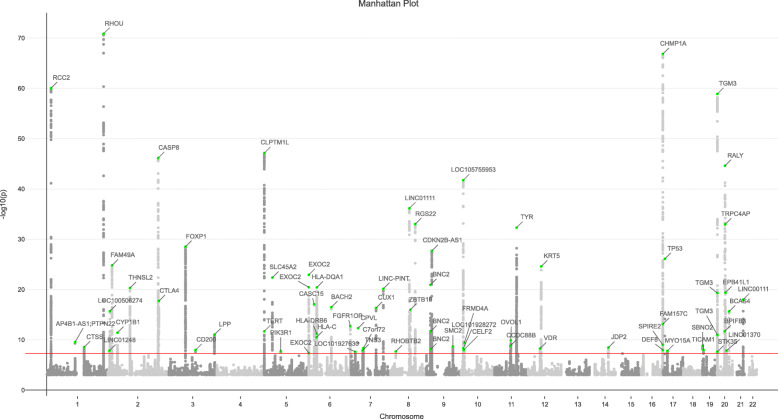
Manhattan plot of basal cell carcinoma GWAS analysis from the UK Biobank. The *x*-axis denotes the chromosome number and position of each variant. The *y*-axis denotes the –log10(*P* value). The 71 independent loci are annotated and highlighted in green (for top SNP in each locus). Those SNPs with *P* > 1 × 10^–3^ have been omitted. The red line denotes the genome-wide significant threshold of *P* < 5 × 10^–8^

We performed GWAS on 7,288,4213 autosomal SNPs with minor allele frequency (MAF) ≥ 0.01 in 17,416 BCC cases and 375,455 controls from the UK Biobank (UKB) (Fig. 1). The estimated SNP-based heritability is 0.170 (*s.e.* = 0.018) on the liability scale, as estimated by LDSC. A total of 71 near-independent SNPs, culminating in 65 loci, significantly associated with BCC (*P* < 5 × 10^−8^) (Additional file 2: Table S1), including 3 new loci not yet described, *PIK3R1*, *RHOBTB2*, and *MYO15A* (Additional file 2: Table S1, bold). In order to identify any potential SNPs masked in GWAS due to LD, we performed conditional and joint association analysis (COJO) and identified a total of 73 jointly significant signals, including 9 additional SNPs which did not reach genome-wide significance in the original GWAS analysis (Additional file 2: Table S2). In particular, 6 COJO signals are located between 89.7~90.1 Mb in chromosome 16 (*MC1R* locus), indicating multiple genetic variants underlying this genomic region (Additional file 1: Figure S2).

### Gene expression analyses reveal 46 functional candidate genes for BCC

In order to identify background genetic factors with the ability to influence or modify the effects of epidermal acquired BCC driver mutations, we chose to interrogate eQTL data from the eQTLGen consortium (*n* = 31,684 blood samples). Biologically, use of a non-epidermal sample source provides optimal opportunity to detect background genetic (and potentially germline) traits and factors that increase susceptibility to BCC. Statistically, we have previously shown that analysing eQTLGen blood eQTL data is more powerful at identifying functional genes than using tissue-specific eQTL data [28], partly due to the significant boost in power that the large eQTL data sample size provides. However, in order to validate that analysis of blood tissue would not affect the validity of the data, we set out to identify the correlation of eQTL effects (*r̂* ) [32]. The ${\hat{r}}_b$ between two independent blood cohorts (eQTLGen and GTEx V7 whole blood) is 0.8344 (s.e. = 0.0051) (Additional file 2: Table S3). Similarly, the ${\hat{r}}_b$ between blood and two GTEx V7 skin samples (skin non-sun exposed and skin sun exposed) are very high, thereby revealing a positive correlation between blood eQTL data and skin tissue.

SMR analysis using our GWAS summary data and eQTL blood data revealed a total of 46 SMR candidate genes whose expression levels were significantly associated with BCC risk (*P*_SMR_ < 3.19 × 10^−6^, i.e. 0.05/*m*_SMR_, with *m*_SMR_ = 15,628 being the total number of SMR tests in eQTLGen dataset) (Table 1). Positive *b*_SMR_ estimates were obtained for 20 SMR genes (Table 1) and negative *b*_SMR_ estimates for 26 SMR genes (Table 1, bold text), linking BCC risk with increased gene expression and decreased gene expression, respectively. HEIDI analysis was performed to filter out the SMR associations (with *P*_HEIDI_ < 0.01) due to LD between the BCC-associated SNPs and the eQTL SNPs, culminating in a refined set of 13 putative causal genes (referred to as SMR-HEIDI genes) (Table 1, asterisk).

**Table 1:** BCC susceptibility functional candidate genes identified via SMR analysis

probeID	Chr	Gene	topSNP	topSNP_bp	A1	A2	Freq	***b***_**GWAS**_	*P*_GWAS_	***b***_**eQTL**_	*P*_eQTL_	***b***_**SMR**_	*P*_SMR_	*P*_HEIDI_
ENSG00000256049	1	***PADI6***	rs6678121	17719986	G	T	0.344	**− 0.097**	9.60E−17	**0.464**	0.00E+00	**− 0.208**	1.80E−16	9.76E−22
ENSG00000179051	1	*RCC2*	rs12035179	17793114	C	T	0.393	**0.080**	3.60E−13	**0.207**	1.19E−148	**0.389**	2.54E−12	8.16E−16
ENSG00000003400	2	***CASP10***	rs13005094	202092561	T	C	0.474	**0.059**	6.50E−08	**− 0.118**	7.77E−50	**− 0.500**	3.82E−07	1.31E−11
ENSG00000064012	2	***CASP8***	rs7560328	202164837	A	C	0.362	**0.130**	3.00E−31	**− 0.207**	7.38E−152	**− 0.627**	2.12E−26	1.90E−09
ENSG00000155749	2	*ALS2CR12*	rs2110690	202185132	G	A	0.502	**− 0.121**	1.40E−28	**− 0.157**	7.92E−88	**0.771**	3.57E−22	2.89E−08
ENSG00000213090	2	***AC007256.5***	rs2540430	202368514	T	A	0.302	**0.105**	2.20E−19	**− 0.072**	3.21E−17	**− 1.456**	7.41E−10	4.43E−05
ENSG00000082146	2	***STRADB***	rs7575721	202256778	C	T	0.321	**0.094**	2.60E−16	**− 0.084**	3.73E−26	**− 1.120**	9.39E−11	1.77E−08
ENSG00000163599	2	*CTLA4*	rs13030124	204694263	A	G	0.430	**0.085**	1.00E−14	**0.147**	2.30E−70	**0.576**	1.35E−12	1.11E−07
ENSG00000049656	5	***CLPTM1L***	rs13170453	1317481	G	A	0.228	**− 0.146**	3.50E−27	**0.090**	5.61E−15	**− 1.617**	2.45E−10	1.37E−05
ENSG00000137265	6	*IRF4*	rs12526822	428486	A	G	0.321	**− 0.098**	2.30E−16	**− 0.070**	1.81E−14	**1.396**	2.13E−08	3.22E−08
ENSG00000196126	6	*HLA-DRB1*	rs9271520	32589771	G	A	0.350	**− 0.092**	2.20E−15	**− 0.483**	0.00E+00	**0.190**	3.97E−15	1.98E−14
ENSG00000237541	6	***HLA-DQA2***	rs9271520	32589771	G	A	0.350	**− 0.092**	2.20E−15	**0.613**	0.00E+00	**− 0.150**	3.09E−15	1.22E−09
ENSG00000204267	6	*TAP2*	rs4148876	32796793	A	G	0.071	**− 0.114**	2.90E−07	**− 0.524**	6.40E−274	**0.218**	3.82E−07	3.88E−04
ENSG00000112182	6	BACH2*	rs72928038	90976768	A	G	0.178	**− 0.124**	3.30E−17	**− 0.290**	9.04E−97	**0.426**	5.29E−15	3.10E−01
ENSG00000071242	6	*RPS6KA2*	rs2757050	167377165	T	G	0.469	**0.056**	2.50E−07	**0.316**	0.00E+00	**0.177**	3.12E−07	1.14E−07
ENSG00000026297	6	*RNASET2*	rs393727	167398632	T	A	0.469	**0.056**	2.20E−07	**0.858**	0.00E+00	**0.065**	2.22E−07	9.81E−06
ENSG00000197146	6	*AL133458.1*	rs408087	167398952	C	T	0.469	**0.056**	2.10E−07	**0.715**	0.00E+00	**0.079**	2.15E−07	6.13E−07
ENSG00000227598	6	*RP1-167A14.2*	rs415987	167395375	G	A	0.469	**0.056**	2.70E−07	**0.304**	0.00E+00	**0.184**	3.38E−07	3.31E−07
ENSG00000112486	6	***CCR6***	rs3093025	167532731	A	G	0.438	**− 0.065**	3.90E−09	**0.208**	4.98E−148	**− 0.311**	9.30E−09	3.16E−04
ENSG00000245025	8	***RP11-875O11.1***	rs2241261	22876739	C	T	0.476	**− 0.056**	4.00E−07	**0.143**	4.56E−73	**− 0.390**	1.05E−06	7.68E−05
ENSG00000173068	9	*BNC2*	rs12350739	16885017	G	A	0.385	**− 0.078**	9.60E−12	**− 0.255**	1.01E−184	**0.304**	3.33E−11	1.72E−03
ENSG00000147883	9	***CDKN2B***	rs2069422	22008026	G	T	0.100	**0.111**	1.60E−10	**− 0.659**	0.00E+00	**− 0.169**	2.34E−10	2.71E−05
ENSG00000136824	9	SMC2*	rs2122576	106870187	C	A	0.395	**0.064**	8.30E−09	**0.212**	1.04E−138	**0.299**	1.97E−08	3.69E−02
ENSG00000236935	11	AP003774.1*	rs479777	64107477	C	T	0.344	**0.068**	2.50E−09	**0.617**	0.00E+00	**0.110**	2.85E−09	1.55E−01
ENSG00000111424	12	VDR*	rs7975232	48238837	C	A	0.480	**− 0.063**	9.00E−09	**0.124**	6.51E−56	**− 0.503**	6.65E−08	2.52E−01
ENSG00000261253	16	***AC137932.6***	rs1078578	89386934	G	A	0.353	**0.075**	2.90E−11	**− 0.119**	9.58E−47	**− 0.630**	1.59E−09	5.89E−05
ENSG00000261118	16	RP11-104N10.1*	rs4785687	89588896	A	G	0.384	**0.097**	2.20E−18	**− 0.060**	1.11E−13	**− 1.605**	1.50E−08	4.58E−02
ENSG00000197912	16	***SPG7***	rs4785686	89587871	C	A	0.417	**− 0.083**	5.60E−14	**0.189**	4.96E−128	**− 0.441**	7.25E−13	1.21E−06
ENSG00000167523	16	***C16orf55***	rs164749	89708224	G	T	0.432	**− 0.061**	2.50E−08	**0.114**	2.28E−47	**− 0.538**	1.96E−07	1.82E−06
ENSG00000185324	16	***CDK10***	rs77651727	89708267	T	C	0.076	**0.096**	1.50E−06	**− 1.419**	0.00E+00	**− 0.067**	1.68E−06	2.09E−04
ENSG00000158792	16	SPATA2L*	rs396742	89768056	G	C	0.422	**− 0.065**	1.00E−08	**− 0.369**	0.00E+00	**0.175**	1.39E−08	1.30E−02
ENSG00000158805	16	*ZNF276*	rs3743859	89846050	T	C	0.412	**0.052**	2.30E−06	**0.244**	6.29E−198	**0.213**	3.07E−06	1.34E−23
ENSG00000204991	16	***SPIRE2***	rs2376879	89884822	G	C	0.292	**− 0.075**	5.40E−10	**0.360**	0.00E+00	**− 0.209**	7.55E−10	1.43E−42
ENSG00000141013	16	***GAS8***	rs45583731	90106364	A	C	0.428	**0.068**	4.40E−10	**− 0.192**	6.97E−91	**− 0.355**	2.50E−09	1.96E−10
ENSG00000141510	17	TP53*	rs35850753	7578671	T	C	0.018	**0.282**	7.00E−15	**− 0.365**	4.04E−21	**− 0.772**	1.93E−09	2.60E−01
ENSG00000091542	17	ALKBH5*	rs2925138	18092509	A	G	0.426	**0.060**	3.80E−08	**0.157**	1.63E−68	**0.383**	1.55E−07	3.76E−02
ENSG00000127666	19	TICAM1*	rs10405449	4821949	T	C	0.372	**− 0.062**	4.40E−08	**0.217**	5.05E−139	**− 0.286**	8.79E−08	5.72E−02
ENSG00000125780	20	***TGM3***	rs214787	2283667	C	T	0.181	**0.214**	3.30E−58	**− 0.357**	0.00E+00	**− 0.599**	7.31E−50	1.55E−08
ENSG00000101421	20	CHMP4B*	rs2626562	32409142	G	A	0.530	**0.053**	1.50E−06	**0.370**	0.00E+00	**0.142**	1.74E−06	7.17E−01
ENSG00000125977	20	***EIF2S2***	rs6142101	32697845	G	A	0.410	**0.052**	2.00E−06	**− 0.243**	7.51E−211	**− 0.215**	2.66E−06	2.07E−17
ENSG00000101460	20	***MAP1LC3A***	rs6059919	33151545	G	T	0.177	**0.132**	3.90E−22	**− 0.642**	0.00E+00	**− 0.206**	1.51E−21	7.05E−04
ENSG00000078804	20	TP53INP2*	rs1884432	33342439	T	C	0.182	**0.127**	5.70E−21	**− 0.203**	1.31E−72	**− 0.626**	8.03E−17	8.15E−02
ENSG00000198646	20	NCOA6*	rs6058112	33322006	G	C	0.179	**0.131**	4.70E−22	**0.208**	1.55E−77	**0.632**	1.01E−17	7.10E−02
ENSG00000131067	20	***GGT7***	rs4911164	33479488	G	C	0.377	**− 0.053**	2.00E−06	**0.201**	1.44E−139	**− 0.266**	3.07E−06	4.36E−14
ENSG00000100991	20	*TRPC4AP*	rs6058166	33656710	C	G	0.394	**0.054**	9.00E−07	**0.589**	0.00E+00	**0.093**	9.40E−07	4.32E−11
ENSG00000100029	22	PES1*	rs737953	30987861	G	C	0.399	**− 0.053**	1.60E−06	**0.174**	1.57E−108	**− 0.307**	2.67E−06	2.08E−01

Genes formatted in bold: decreased gene expression linked to increased risk of BCC. Genes not in bold: increased gene expression linked to increased risk of BCC. Genes denoted by asterisk (*): genes that passed the HEIDI test. Columns: Probe ID; Probe chromosome; Gene, gene name; Probe_bp, probe position; topSNP, SNP ID; topSNP_bp, top eQTL position; A1, effect allele; A2, alternative allele; Freq, frequency; *b*_GWAS_, GWAS effect; *P*_GWAS_, GWAS *P* value; *b*_eQTL_, eQTL effect; *P*_eQTL_, eQTL *P* value; *b*_SMR_, SMR effect; *P*_SMR_, SMR *P* value; *P*_HEIDI_, HEIDI *P* value

### DNA methylation analyses define 5 loci that exhibit both genetic and methylation regulatory mechanisms linked to BCC susceptibility

**Fig. 2:**
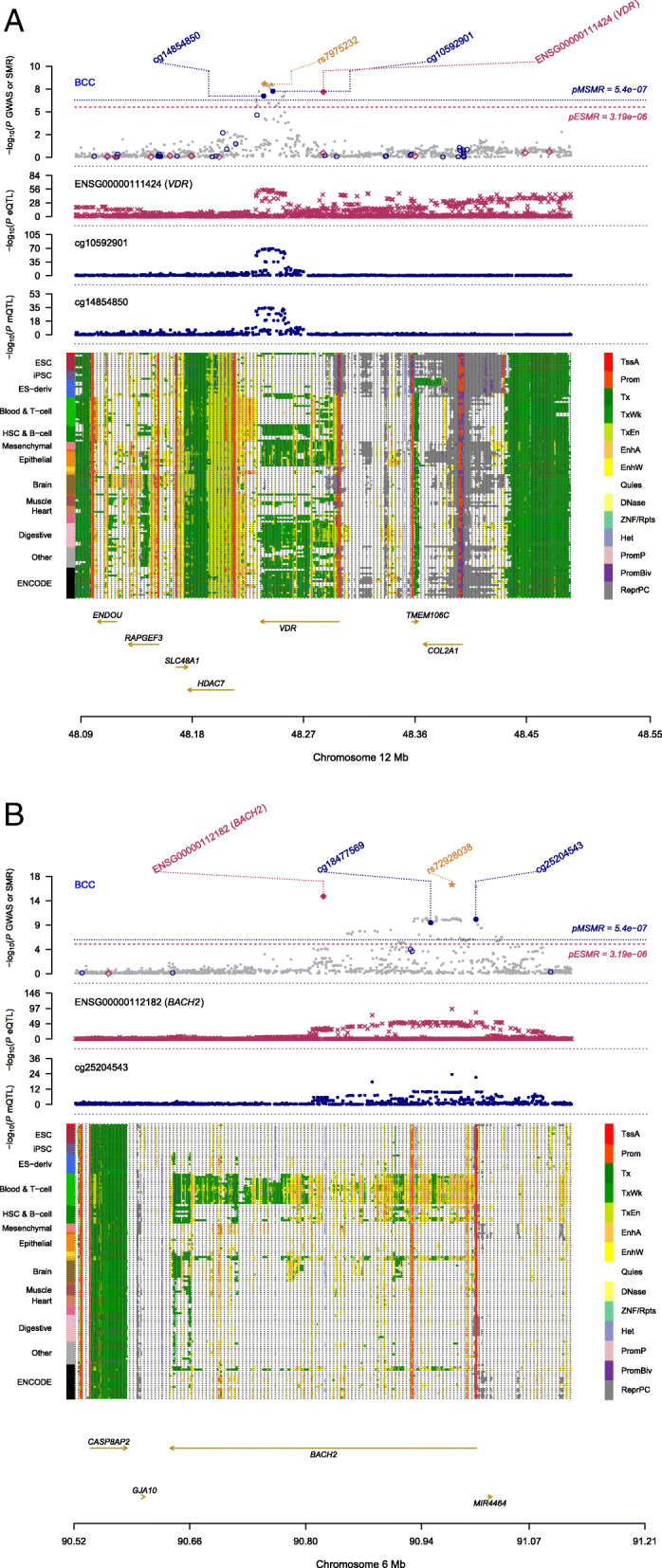
Integration of GWAS, eQTL, and mQTL data for VDR and BACH2 genes. **a** –log_10_(*P* value) of SNPs from BCC GWAS analysis. Gene expression and methylation probes are annotated by red diamonds and blue circles, respectively. Solid diamonds and circles denote probes that passed the HEIDI filtering test (*P*_HEIDI_ > 0.01). Yellow star highlights the top cis-eQTL SNP (rs7975232). **b** –log_10_(*P* value) of SNP association with gene expression (probe ENSG00000111424 tagging VDR). **c** –log_10_(*P* value) of SNP association with methylation (DNAm probe cg14854850). **d** The upper panel shows 25 chromatin state annotations under the genomic region (e.g. promoters and enhancers, annotated by colours on the right bar) from the Roadmap Epigenomics Mapping Consortium. Each row denotes one of the 127 samples with different tissue and cell types (each type annotated by colours on the left bar). The lower panel shows the genes underlying this region and their genomic positions. **e** –log_10_(*P* value) of SNPs from BCC GWAS analysis, as described in **a**. Yellow star highlights the top cis-eQTL SNP (rs7298038). **f** –log_10_(*P* value) of SNP association with gene expression (probe ENSG00000112182 tagging BACH2). **g** –log_10_(*P* value) of the SNP association with methylation (DNAm probe cg25204543). **h** The upper panel shows 25 chromatin state annotations as described in **d**. The lower panel shows the genes underlying this region and their genomic positions

**Fig. 3:**
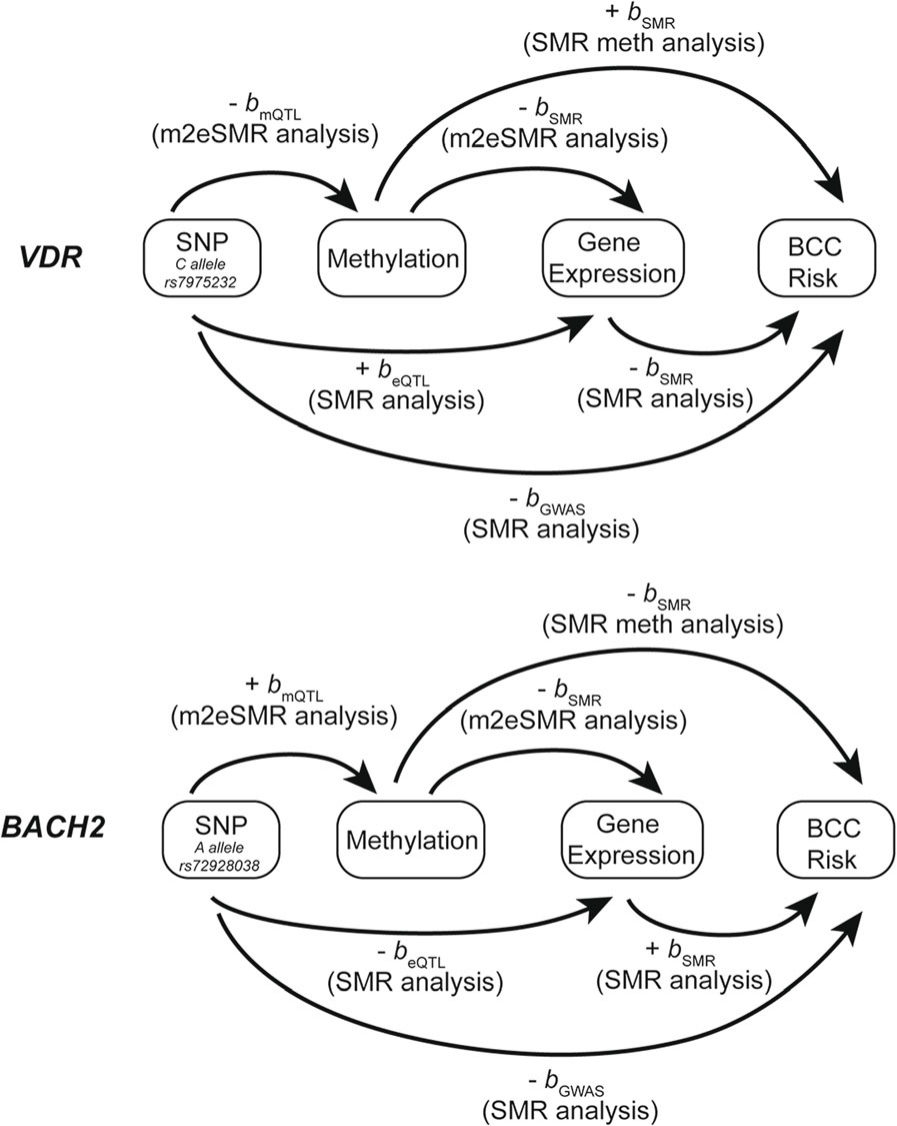
Diagrammatic summary of all genome-wide estimates for VDR and BACH2 genes. Congruent estimates for *b*_*GWAS*_, *b*_*eQTL*_, *b*_*mQTL*_, and *b*_*SMR*_, revealing strength and power of the methodology used in this study. *b*_*GWAS*_ denotes the effect of variant-phenotype association. *b*_*eQTL*_ denotes the effect of variant-expression association. *b*_*mQTL*_ denotes the effect of the variant on the methylation level. *b*_*SMR*_ denotes the effect of gene expression on the disease risk in the SMR analysis

In order to identify epigenetic regulatory signals associated with BCC susceptibility, we focused on methylation QTL (mQTL) data in blood sample (referred to as mSMR analysis) and identified 54 DNA methylation (DNAm) probes (located in 18 independent loci) that were significantly associated with BCC (*P*_SMR_ < 5.40 × 10^−7^ [*m*_SMR_ = 92,557] and *P*_HEIDI_ ≥ 0.01) (Additional file 2: Table S4). By performing an SMR analysis that genetically links DNAm to gene expression (m2eSMR analysis), we identified 41 DNAm sites associated with gene expression. Twenty-seven of the DNAm sites, all located within chromosome 16, were found to associate with seven functionally relevant genes (Additional file 2: Table S5). However, only *SPATA2L* and *RP11-104N10.1* passed the eSMR HEIDI test (*P*_HEIDI_ ≥ 0.01) (Table 1). These data, in addition to the COJO analysis findings (Additional file 2: Table S2), indicate that multiple causal variants reside in this region of the genome. A total of 5 loci (*BACH2*, *VDR*, *STRADB*, *SPG7*, and *HLA-DRB1*/*DQA2*) were identified to exhibit genome-wide significance in both eQTL and DNAm analyses and significant association between DNAm and eQTL (*P*_DNAm->eQTL_ < 1 × 10^−5^, Additional file 2: Table S5), indicating genetic and methylation regulatory mechanisms driving BCC susceptibility. The combined GWAS, eQTL, and mQTL locus plots of the *BACH2* and *VDR* loci (Fig. 2) and assembly of all the omics level estimates for both genes (Fig. 3, Table 1, Additional file 2: Table S4-S5) are all congruent, revealing the strength of our methodology. In particular, one DNAm site (cg25204543) located in the promoter region of *BACH2* (Fig. 2) passed the most stringent thresholds in SMR and HEIDI, indicating a potential regulatory mechanism driving BCC risk. The A allele of variant rs72928038 showed decreasing effect on the expression level of *BACH2* via upregulating the methylation level of cg25204543 (located in the promoter region of *BACH2*), and the increased expression of *BACH2* was associated with higher BCC risk.

### Protein interaction networks of BCC susceptibility associations reveal a highly connected system

Local FI networks were constructed by inputting each of the 71 GWAS hits (using nearest genes) and the 46 SMR candidate genes into the Reactome database. The resulting FI networks represent a global overview of the protein-protein interactions, representing biological functions such as binding, activation, translocation, degradation, classical biochemical events, and catalyst reactions. The generated GWAS-FI network consists of 42 GWAS nearest gene (proteins) and 29 protein interactors (Additional file 1: Figure S3). Remarkably, it presents as one large interconnected protein network with UBC and P1K3R1 acting as the most highly connected nodes within the network (Additional file 1: Figure S3, red hubs). Pathway analysis of the GWAS-FI network revealed cell cycle and cell death processes, with particularly strong enrichment of immune regulation processes, with 11 of the top 20 pathways (Additional file 2: Table S6) and 4 of the top 10 GO-Biological processes (Additional file 2: Table S7) linked to immune system function.

**Fig. 4:**
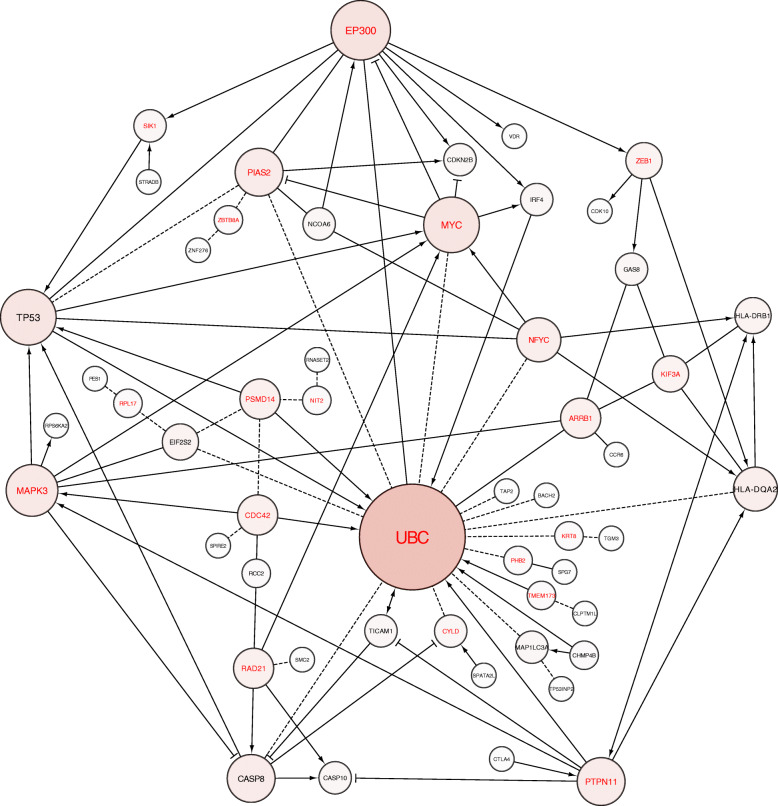
Functional interaction network of the protein-coding genes identified via SMR analysis. Genes listed in black indicate SMR proteins. Genes listed in red indicate protein interactors. In this network, "→" indicates activating/catalysing, “-|” inhibition, “---” predicted FIs, and “-” FIs extracted from complexes or inputs

The SMR-FI network also formed an interconnected multidimensional network, consisting of 32 SMR genes and 20 protein interactors (Fig. 4). Similarly, pathway analysis of the SMR-FI network revealed cell cycle and cell death processes, with particularly strong enrichment of immune regulation processes, with 9 of the top 20 pathways (Additional file 2: Table S8) and 14 of the top 50 GO-Biological processes linked to immune system activity (Additional file 2: Table S9). These data indicate strong enrichment for immune regulation genes within both the GWAS and SMR-FI networks. We subsequently queried PubMed databases for each of the 46 SMR candidate genes used to create the FI network and confirmed enrichment of three predominant biological processes: cell cycle regulation, cell death, and immune regulation (Additional file 2: Table S10). Interestingly, 11/46 of the SMR and 5/13 SMR-HEIDI genes are associated with regulatory T cell (T_Reg_) activity (Additional file 2: Table S10).

### Blood and skin gene expression analyses reveal common functional candidate genes

Although blood remains the most accessible source for large-scale transcript profiling, thus ensuring adequate power to detect eQTL, it is equally important to investigate the potential of tissue-specific changes in gene expression. We therefore explored the degree of tissue-specific eQTL overlap between blood and skin samples. We performed SMR analysis using our GWAS summary data and eQTL data from sun exposed skin (sun exposed lower leg, *n* = 605), non-sun exposed skin (sun not exposed suprapubic, *n* = 517), and a smaller cohort of whole blood (*n* = 369) from the GTEx V7 dataset. A total of 25 significant SMR hits ( *P*_SMR_< 7.63 × 10^−6^, i.e. 0.05/6557) were identified in sun exposed skin and 21 significant SMR hits (*P*_SMR_ < 9.52 × 10^−6^, i.e. 0.05/5252) in non-sun exposed skin (Table 2 and Additional file 2: Table S11) culminating in a total of 12 unique skin-specific SMR genes (Table 2). Whole blood revealed 20 significant SMR hits (*P*_SMR_ < 1.12 × 10^−5^, i.e. 0.05/4459), 15 of which overlapped with the eQTLGen results (Table 2 and Additional file 2: Table S11). Although the sample size of blood tissue in GTEx dataset (*n* = 369) is much smaller than that of eQTLGen dataset (*n* = 31,684), the *b*_SMR_ estimates for the 15 overlapping SMR hits show very high consistency (Pearson’s correlation *r* is 0.92, *s.e.* = 0.05) (Additional file 2: Table S12), indicating the *b*_SMR_ estimates are robust for the same tissue from different datasets. Only 7 SMR genes are common among the four datasets analysed (Table 2). This is likely attributable to sample size (SMR only selects probes with a *P*_eQTL_ < 5 × 10^−8^), the different number of probes used for SMR analysis across the datasets (eQTLGen = 15,652, GTEx = 4459~6557) and sampling variations. For example, of the 46 genes significant in eQTLGen SMR, only 22 of them had a *P*_eQTL_ < 5 × 10^−8^ in the GTEx dataset. The seven common SMR genes span the three predominant biological processes identified in the eQTLGen-SMR gene list: immune regulation, cell cycle regulation, and cell death (Additional file 2: Table S10). The identification of 24 SMR genes unique to the eQTLGen dataset analysis highlights the statistical power gained by performing SMR analyses on tissues with very large sample size and is consistent with our previous studies [28, 32].

**Table 2:** Overview of blood and skin SMR analyses detailing tissue-specific and common BCC susceptibility genes

**Tissue**	**Number of SMR genes**	**Sample size**	**Number of probes tested**	***P*** **value threshold of multiple correction**
Blood (eQTLGen)	46	31,684	15,652	3.19E−06
Skin_Sun_Exposed (GTEx)	25	243	6557	7.63E−06
Skin_Not_Sun_Exposed (GTEx)	21	216	5252	9.52E−06
Whole_Blood (GTEx)	20	360	4459	1.12E−05
Overall number of unique elements	63	/	/	/
**Tissue**	**Number of overlapping genes**	**Gene name**		
Blood (eQTLGen) Skin_Not_Sun_Exposed (GTEx) Skin_Sun_Exposed (GTEx) Whole_Blood (GTEx)	7	*SPIRE2*, *CDK10*, *HLA-DRB1*, *HLA-DQA2*, *AL133458.1*, *RNASET2*, *CASP8*		
Blood (eQTLGen) Skin_Not_Sun_Exposed (GTEx) Skin_Sun_Exposed (GTEx)	1	*ALS2CR12*		
Blood (eQTLGen) Skin_Sun_Exposed (GTEx) Whole_Blood (GTEx)	1	*SPATA2L*		
Skin_Not_Sun_Exposed (GTEx) Skin_Sun_Exposed (GTEx) Whole_Blood (GTEx)	2	*HLA-DOB*, *HLA-DRB6*		
Blood (eQTLGen) Skin_Sun_Exposed (GTEx)	3	*CLPTM1L*, *RP11-104N10.1*, *ALKBH5*		
Blood (eQTLGen) Skin_Not_Sun_Exposed (GTEx)	3	*SMC2*, *TGM3*, *NCOA6*		
Blood (eQTLGen) Whole_Blood (GTEx)	7	*RCC2*, *SPG7*, *PADI6*, *SPATA33*, *MAP1LC3A*, *CDKN2B*, *AP003774.1*		
Skin_Not_Sun_Exposed (GTEx) Skin_Sun_Exposed (GTEx)	7	*DBNDD1*, *TCF19*, *ASIP*, *PSORS1C3*, *HLA-DQA1*, *KRT6C*, *POU5F1*		
**Tissue**	**Number of unique genes**	**Gene name**		
Blood (eQTLGen)	24	*VDR*, *RP1-167A14.2*, *GGT7*, *IRF4*, *EIF2S2*, *TAP2*, *TRPC4AP*, *BNC2*, *AC007256.5*, *RP11-875O11.1*, *GAS8*, *RPS6KA2*, *ZNF276*, *CCR6*, *PES1*, *BACH2*, *CASP10*, *TP53*, *TP53INP2*, *CTLA4*, *AC137932.6*, *CHMP4B*, *STRADB*, *TICAM1*		
Skin_Sun_Exposed (GTEx)	4	*FANCA*, *SEMA6C*, *CTSS*, *URAHP*		
Skin_Not_Sun_Exposed (GTEx)	1	*FGFR1OP*		
Whole_Blood (GTEx)	3	*CHMP1A*, *HLA-DRB9*, *SPAG1*		

Table revealing the number of SMR genes, sample size, number of probes tested, and multiple correction threshold in each tissue and the SMR genes unique to each tissue-gene dataset and list of SMR genes common among datasets. In the eQTLGen dataset, the gene name *SPATA33* is aliased as *C16orf55*. To ease gene comparison analyses, *C16orf55* was changed to *SPATA33* in the eQTLGen results

## Discussion

GWAS have provided a powerful approach to the dissection of the genetic components of complex traits. However, the complicated linkage disequilibrium structure of the human genome and the observation that genetic variants can affect phenotype via distant regulation of gene expression recue the power of GWAS alone to identify the specific genes that underlie these complex traits. Here, we used the power of the UK Biobank to perform a GWAS of genetic susceptibility to BCC as the platform, upon which we built an integrated approach of SMR analysis focused on both eQTL and mQTL, followed by protein functional interaction (FI) network analysis. Consequently, we not only defined a number of new BCC susceptibility loci but more significantly identified three dominant processes underlying that susceptibility—cell death (25/46 SMR genes), cell cycle (23/46 SMR genes), and immune regulation (20/46 SMR genes). Given that control of cell cycle and cell death is well characterised in the biology of BCC formation, we interrogated our SMR functional susceptibility gene list and FI networks to dissect how genetic susceptibility to BCC is influenced by inherited background immune traits. Reduced expression of HLA is one key mechanism by which tumours escape host immune surveillance [33], and our SMR analyses identified decreased expression of *HLA-DQA2* is linked to increased BCC risk. *TAP2*, another SMR gene, localises to the MHC class II region and plays a pivotal role in immune surveillance, with polymorphisms linked to the susceptibility of various autoimmune disorders [34–36] and various neoplasms [37–39].

Of particular interest, our integrative approach revealed a strong enrichment of BCC susceptibility genes (24% of SMR and 38% of SMR-HEIDI candidate genes) involved in regulatory T cell (T_Reg_) activity. These include previously identified GWAS loci including CTLA4 [10], IRF4 [12], VDR [8], and SMC2 [10]. T_Regs_ are essential for maintaining immune homeostasis by limiting effector T cell activity against foreign antigens. A particularly interesting T_Reg_-BCC susceptibility gene identified here as a GWAS locus and an SMR-HEIDI eQTL and mQTL gene is *BACH2*. BACH2 has been linked to B cell lymphoma, CML, and stomach cancer [40–42] and has also been shown to be required for efficient formation of T_Regs_ [43]. Consequently, Bach2-deficient mice exhibit markedly impaired tumour growth due to increased effector T cell activation and a reduction in T_Regs_ [44]. Our discovery that increased *BACH2* expression correlates with increased risk of BCC (+*b*_SMR_), alongside identification of a BCC-associated methylation site in the promoter of *BACH2* which increases gene expression, suggests a molecular mechanism whereby elevated levels of BACH2 promote tumour immunosuppression by attenuating effector T cells. In support of this, BACH2 was recently shown to specifically restrain TCR-driven T_Reg_ activation and actively drive T_Reg_ quiescence [45], thereby indicating BACH2 functions to promote tumour immunosuppression by both upholding a durable T_Reg_ precursor pool and also maintaining T_Regs_ functionally quiescent. Similarly, interrogation of our GWAS and SMR FI networks also revealed strong enrichment of protein hubs linked to T_Regs_. Conditional deletion of EP300, a highly connected protein interactor in both the GWAS-FI (exhibiting 16 interactions) and SMR-FI network (exhibiting 9 interactions), results in impaired T_Reg_ suppressive function and reduced tumour growth [46]. PTPN11 acts as a protein hub within the SMR-FI network, exhibiting a total of 7 neighbouring nodes, 4 of which are T_Reg_-associated SMR genes. Myc, another protein hub in the SMR-FI network whose role in cancer has been the focus of intense study over many years, is directly connected to 2 of the 9 T_Reg_ genes represented on the network and can be connected to a total of 8 T_Reg_ genes via one linker protein. Taken together, these data reveal that background genetic factors regulating T_Regs_ immune function act to predispose an individual to BCC.

The mechanism by which BCC susceptibility genes involved in T_Reg_ activity likely function to predispose an individual to BCC is via regulating the tumour microenvironment (TME). The TME is a complex system consisting of tumour cells, endothelial/vascular cells, stroma, and immune cells, and evidence indicates that the interplay between immune cells and other components of the TME largely determines tumour cell survival and disease progression [47]. Recent studies have shown consistency in the TME of immune cells across BCC patients [48], whereas other studies have reported a high proportion of BCCs (82%) present with expression of immune checkpoint proteins on the tumour-infiltrating lymphocytes located in the TME [49]. All these data support how changes in gene expression, as defined by innate genetic predisposition, that produce an immune evasive TME can contribute to the susceptibility of an individual to BCC tumour formation.

Another principle finding of our analyses is the identification of functional candidate genes that were previously reported GWAS hits. Using SMR and HEIDI analysis [16], we demonstrated a direct correlation between GWAS SNP association and changes in gene expression. Importantly, the directions of gene expression change (whereby positive *b*_SMR_ estimates represent increased gene expression linked increased risk of disease and negative *b*_SMR_ values represent decreased gene expression linked increased risk of disease) are all consistent with their biological function as reported in the literature. Interrogation of our GWAS-FI and SMR-FI network revealed a host of previously described processes linked to BCC susceptibility including “cellular response to UV”, “apoptotic process”, and “DNA damage response, signal transduction by p53”. Skin-specific processes, however, such as “melanin biosynthetic process”, “keratinisation”, “positive regulation of hair cycle”, and “hair follicle placode formation” were only present in the GWAS-FI network. Given we have shown a high degree of correlation ${\hat{r}}_b$ between blood and skin eQTL effects, it is unlikely that the GWAS loci contributing to these skin-specific processes failed to progress to SMR genes as a direct consequence of interrogating blood eQTLGen data. We did, however, identify both blood-specific and skin-specific SMR genes when analysing the degree of tissue-specific eQTL overlap using smaller eQTL cohorts. Hence, it remains to be determined whether skin-specific GWAS loci could be identified as functional candidate SMR genes upon access to a large-scale transcript profiling skin dataset, providing adequate power to detect eQTL.

## Conclusions

Our data provide important insights into the relationship between disease and host genotype in the most common form of human cancer. Additionally, given the high prevalence of HH pathway activity in BCC, the discovery of genes contributing significantly to polygenic risk illustrates a conceptual framework whereby host genotype is critical for the development of cancer even in the presence of clear somatic oncogenic drivers. Clinically, our data suggest that maintenance of strong cutaneous immunity be incorporated into current BCC prevention strategies/guidelines, thereby strengthening the likelihood of mounting an immune response to tumour antigens in the early stages of cancer formation. Taken together, our association and candidate gene studies have unearthed risk variants that function in a highly interconnected regulatory network and identify potential avenues for intervention.

## Supplementary Information

**Additional file 1: Supplementary Figures**. **Figure S1**. Gender and age demographics/distribution of UK Biobank derived BCC cases and controls. **Figure S2**. Regional plots of *MC1R*. **Figure S3**. FI network for the protein-coding ‘nearest-genes’ identified by GWAS analysis.

**Additional file 2: Supplementary Tables**. **Table S1**. Independent common variants associated with BCC in GWAS analysis at *P*-value < 5E-8. **Table S2**. Common variants identified by GCTA-COJO analysis of BCC at *P* < 5E-8 . **Table S3**. Quantification of the correlation of eQTL effects (*r̂* ) between blood and skin samples. **Table S4**. BCC-associated CpG methylation sites via SMR analysis of the GWAS meta-analysis and mQTL data. **Table S5**. Mapping the BCC-associated CpG methylation sites to the BCC-associated genes via SMR analysis using the eQTLGen eQTL data. **Table S6**. BCC GWAS-FI network Pathway Analysis. **Table S7**. BCC GWAS-FI network GO-Process Analysis. **Table S8**. BCC SMR-FI network Pathway Analysis. **Table S9**. BCC SMR-FI network GO-Process Analysis. **Table S10**. Pubmed search of SMR-HEIDI candidate genes biological processes. **Table S11**. BCC-associated genes identified via SMR analysis using GTEx and eQTLGen eQTL data. **Table S12**. Pearson’s correlation of GTEx and eQTLGen eQTL data. This excel file contains 11 supplementary tables, related to the main text.

## Data Availability

The datasets supporting the conclusions of this article are included within the article and its additional files. Summary statistics of the BCC GWAS can be accessed in GWAS Catalog via ftp://ftp.ebi.ac.uk/pub/databases/gwas/summary_statistics/GCST90013410 [50]. Summary statistics of the BCC GWAS are also available at http://fastgwa.info/share/bcc-paper/.

## Abbreviations

TME: Tumour Microenvironment
BCC: Basal cell carcinoma
UV: Ultraviolet
HH: Hedgehog
GWAS: Genome-wide association study
LD: Linkage disequilibrium
eQTL: Expression quantitative trait locus
mQTL: Methylation quantitative trait locus
SNP: Single nucleotide polymorphism
SMR: Summary-data-based Mendelian Randomisation
HEIDI: HEterogeneity In Dependent Instruments
COJO: Conditional and joint association analysis
T_Reg_: Regulatory T cell
