# Supplementary material for: Genetic and functional interaction network analysis reveals global enrichment of regulatory T cell genes influencing basal cell carcinoma susceptibility
Source: Genome Med. 2021 Feb 6;13:19. doi: 10.1186/s13073-021-00827-9 (PMC7866769; doi:10.1186/s13073-021-00827-9)
Supplement: Supplementary file 1 — Additional file 1: Supplementary Figures. Figure S1. Gender and age demographics/distribution of UK Biobank derived BCC cases and controls. Figure S2. Regional plots of MC1R. Figure S3. FI network for the protein-coding ‘nearest-genes’ identified by GWAS analysis. [file 13073_2021_827_MOESM1_ESM.pdf]

BCC	Count	Male/female ratio	Mean of Age	S.D. of Age
Case	17,416	0.501	63.0	6.59
Control	375,455	0.468	58.1	8.06

S.D. = standard deviation

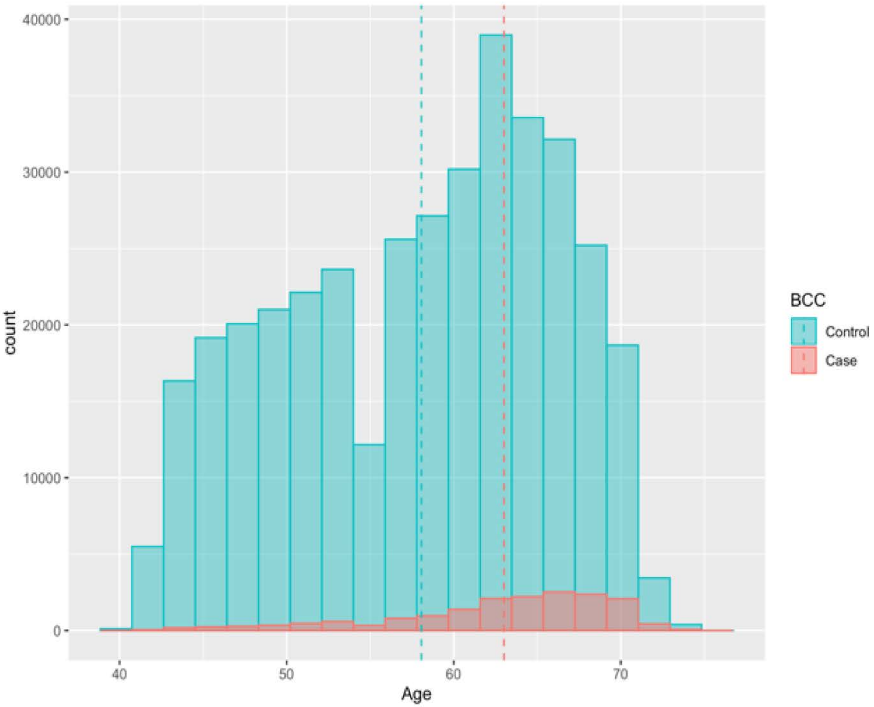

Figure S1. Gender and age demographics/distribution of UK Biobank-derived BCC cases and controls. Age distribution histogram plot. x-axis is the age of each participant. y-axis is the count of participants in each interval. Blue bars represent controls. Red bars represent BCC cases. The two vertical dashed lines represent the mean age of BCC and controls.

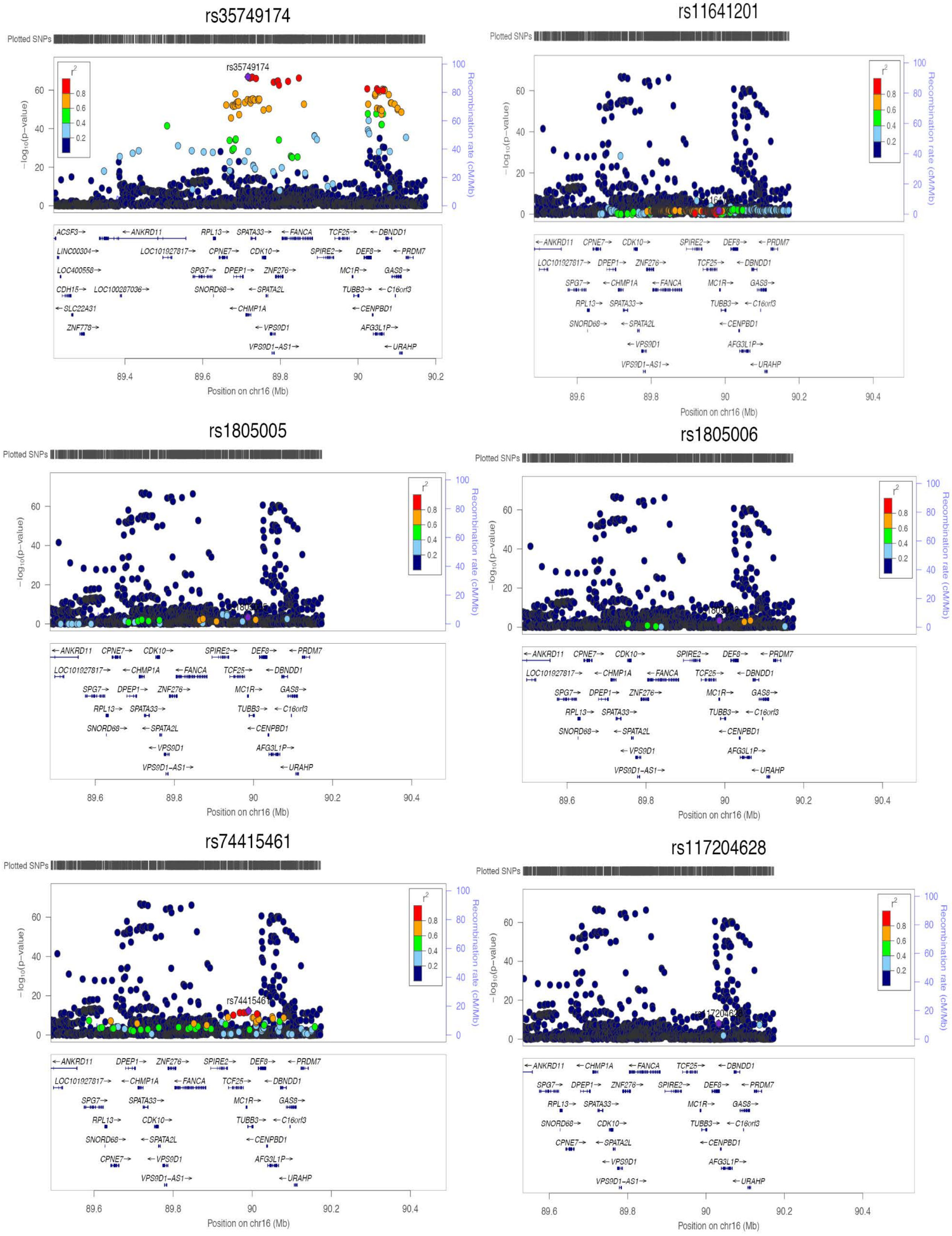

Figure S2. Regional plots of MC1R. Regional plots of MC1R with 6 jointly significant ($P_{COJO} < 5E^{-8}$) SNP as proxy SNP. Each point indicates a SNP in the GWAS with its P value (on the y-axis -log10 scale) against the genomic position (NCBI Build 37). In each plot, the proxy SNP is annotated in purple and the color of all other SNPs indicate the LD r2 estimate with the proxy SNP. The scale of the LD r2 estimate is shown in the legend on the top left/right region in each plot. Recombination rates are calculated based on Phase I 1000 Genomes, and gene annotation information at the bottom are obtained from the UCSC genome browser.

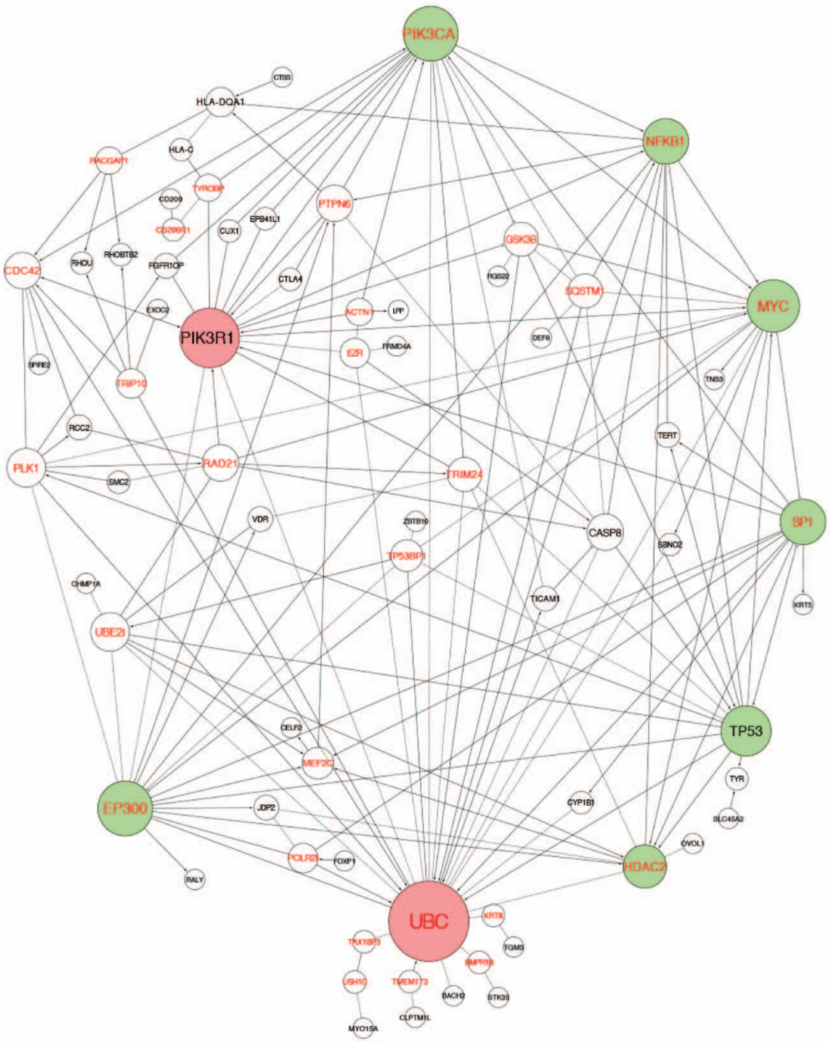

Figure S3. FI network for the protein-coding 'nearest-genes' identified by GWAS analysis. Genes listed in Black indicate GWAS identified loci. Genes listed in Red indicate protein interactors. In this network, "->" indicates activating/catalyzing; "-|" inhibition; "---" predicted FIs and "-" FIs extracted from complexes or inputs.
